# Set up from the beginning: The origin and early development of cassava storage roots

**DOI:** 10.1111/pce.14300

**Published:** 2022-03-30

**Authors:** Anna Vittoria Carluccio, Laure C. David, Joelle Claußen, Marco Sulley, Seun Raheemat Adeoti, Toyin Abdulsalam, Stefan Gerth, Samuel C. Zeeman, Andreas Gisel, Livia Stavolone

**Affiliations:** ^1^ International Institute of Tropical Agriculture Ibadan Nigeria; ^2^ Institute for Sustainable Plant Protection, CNR Bari Italy; ^3^ Department of Biology Institute of Molecular Plant Biology, ETH Zurich Zurich Switzerland; ^4^ Fraunhofer‐Institut für Integrierte Schaltungen IIS Fürth Germany; ^5^ Institute for Biomedical Technologies, CNR Bari Italy

**Keywords:** manioca, rhizogenesis, root bulking, root development, X‐ray computed tomography

## Abstract

Despite the importance of storage root (SR) organs for cassava and the other root crops yield, their developmental origin is poorly understood. Here we use multiple approaches to shed light on the initial stages of root development demonstrating that SR and fibrous roots (FR) follow different rhizogenic processes. Transcriptome analysis carried out on roots collected before, during and after root bulking highlighted early and specific activation of a number of functions essential for root swelling and identified root‐specific genes able to effectively discriminate emerging FR and SR. Starch and sugars start to accumulate at a higher rate in SR before they swell but only after parenchyma tissue has been produced. Finally, using non‐destructive computed tomography measurements, we show that SR (but not FR) contain, since their emergence from the stem, an inner channel structure in continuity with the stem secondary xylem, indicating that SR derive from a distinct rhizogenic process compared with FR.

## Summary Statement

Cassava storage roots are not developing from fibrous roots but follow a distinct rhizogenic process, undescribed before. The key differences are detected starting from root emergence, in gene expression profiles, morphology and anatomy.

## 1 INTRODUCTION

Producing enough food, in a sustainable manner, to meet the needs of an increasing global population is one of the greatest challenges we are confronted with nowadays. Cassava (*Manihot esculenta*) is one of the most important staple food crops providing nutritional security to over 800 million people worldwide. With its storage roots (SR) consisting mostly of starch (up to 90% dry weight; Montagnac et al., 2009), and its resilience to heat and drought stresses, cassava can efficiently respond to critical present and future climate change challenges. Despite 6000 years of cassava domestication (Bredeson et al., 2016) and the relevance of its roots as staple food for millions of people in tropical and subtropical areas, relatively little is currently known about the mechanism of SR formation and starch accumulation of this important crop, and of root crops in general (Hershey et al., 2012; Jammer et al., 2020; Villordon et al., 2014).

Tuberous root development is a complex process involving intense crosstalk among phytohormones, metabolites and regulation of gene transcription to manage cell division and elongation for radial growth and starch accumulation (Li et al., 2016; Utsumi et al., 2020). The anatomical process of SR formation has been well described in classical studies at the light microscopy resolution level (Kokubu, 1973; McCormick, 1916; Togari, 1950; Wilson & Lowe, 1973). Higher activity of the vascular cambium, achieved through cell divisions and expansions in the primary and secondary meristems, lead to an expansion of the root diameter (Togari, 1950), which exhibits a lower degree of lignification in the stele. First, there is rapid division of cambium cells along the root axis. This is followed by vascular cambium production of numerous parenchymatous tissues, resulting in the thickening of the root diameter needed to accumulate large quantities of starch (Naconsie et al., 2016).

Based on the observation that cassava SR start to swell in a bunch of fine‐branched apparently undifferentiated fibrous roots (FR), they are assumed to develop from FR upon receiving one or more specific, but unknown signals (Alves, 2009; El‐Sharkawy, 2004). Many questions remain open regarding this hypothesis, for example, how and why certain FR are selected to become SR, and which (and when) the signal(s) are delivered to (or activated in) the bulking roots.

The mechanism of storage‐organ growth has recently been investigated in various root crops using different molecular, microscopy and metabolite measurement technologies. Up‐regulation of sucrose (Zhang et al., 2017), starch and storage protein biosynthesis, accompanied by delignification (Firon et al., 2013; Sun et al., 2015), cell wall and tissue functional differentiation (Ding et al., 2020; Firon et al., 2013; Kuznetsova et al., 2020) and phytohormone activation (Sojikul et al., 2015) are the major modifications identified in the thickening transition stage and final development into SR. Thus, even though differences between FR and SR have been identified, the earliest stages of SR development have not been studied, particularly at the molecular level, in any of the root crops, and the hypothetical signals changing an FR into an SR have yet to be discovered.

In this study, we used systematic approaches to study the functional differentiation between FR and SR. We provide evidence of different genetic, morphologic and metabolic behaviours of FR and SR starting from their emergence from a planting stem and, therefore, earlier before SR swelling. Non‐destructive X‐ray computed tomography (CT) analysis confirmed the morphological differences during the root emergence stages. Understanding the regulatory processes and the underlying genetic components controlling root growth, and thereby root system architecture, is not only a fascinating question of basic plant biology but also essential for understanding cassava and all other root crops productivity, and is key to breed and engineer better‐performing plants with a potential to feed the future world population in the face of global climate change.

## 2 MATERIALS AND METHODS

### 2.1 Planting and sample materials

The study was conducted at the International Institute of Tropical Agriculture in Ibadan, Nigeria. Freshly *M. esculenta* (cassava) cut stems of the TMEB419 genotype as well as KALESO, TMEB14, TMS‐IBA090576 genotypes, obtained from the IITA Genebank collection, were used as planting material for the study. Trials were laid out in a randomized complete block design with three replications. Healthy stem cuttings, each 25 cm in length were vertically planted in a flat seedbed at a spacing of 1 × 1 m. Samples of FR, potential FR (PFR), SR, potential SR (PSR), source leaves (SOL) and sink leaves (SIL) were obtained from single or multiple organs as further specified. Samples were separated in different biological replicates, cut in small pieces, mixed and snap‐frozen in liquid N_2_.

### 2.2 RNA extraction and quantitative real‐time PCR (RT‐qPCR)

RNA was extracted from samples of SIL, SOL and both PFR/FR and PSR/SR tissue by combining cetyl trimethylammonium bromide (CTAB)‐extraction method and spin‐column‐based purification methods. Total nucleic acid was extracted by using the CTAB (2% CTAB, 2% PVP‐40, 20 mM Tris–HCl, pH 8.0, 1.4 M NaCl, 20 mM ethylenediaminetetraacetic acid) extraction protocol as described by Li et al. (2008) with some modifications. Approximately 500 mg samples were ground in liquid nitrogen and mixed with 1 ml pre‐heated CTAB extraction buffer. Samples were incubated at 65°C for 15 min with intermittent vortexing, then subjected to centrifugation (16 000 *g* at 4°C for 5 min). The supernatant was mixed with an equal volume of cold chloroform: isoamyl alcohol (24:1) before centrifugation (16 000 *g* at 4°C for 10 min). The supernatant was added to 0.6 volumes of cold isopropanol and gently mixed to precipitate the nucleic acids. The pellet was washed with 70% ethanol, air‐dried and dissolved in nuclease‐free water. After DNaseI treatment, the resulting RNA was cleaned up using the kit RNA Clean & Concentrator™ (Zymo Research) according to the manufacturer's instructions.

The RNA concentration in the samples was normalized based on Qubit assays (Invitrogen, Thermo Scientific) and gel observations, and 1 µg of quality‐confirmed RNA was subjected to complementary DNA (cDNA) synthesis as follows. Reverse transcription was completed with 1 µg of total RNA in 20 µl. First‐strand cDNA synthesis was performed using the M‐MuLV Reverse Transcriptase (New England Biolabs) according to the manufacturer's instructions. The reaction contained 1 µg of RNA, 2 µl of 10× RT random primer and 1 µl of 10 mM dNTPs. The mixture was denatured at 65°C for 5 min and placed on ice immediately. Next, 4.4 µl of the RT master mix (consisting of 2 µl 10× RT Buffer, 1 µl of M‐MuLV RT, 0.2 µl of RNasin) were added and the mixture incubated at 25°C for 5 min, at 42°C for 1 h, and 65°C for 20 min. The cDNA synthesized served as a template for qPCR amplification of specific target genes.

Briefly, qPCR reactions containing 10 ng of cDNA, 400 nM of each forward and reverse primers and Luna® Universal qPCR Master Mix (New England Biolabs®) were combined in a total volume of 12.5 µl. Three technical replicates per sample were used except for single plants (Figure 7) where we used four technical replicates. The reactions were conducted in a LightCycler® 480 Instrument (Roche) using the following cycling profile: 10 s at 95°C, followed by 40 cycles of 3 s at 95°C and 30 s at 60°C. Data obtained were converted into relative gene expression using the $2^{‐\Delta \Delta C_{t}}$ method corrected for the PCR efficiency of each amplicon using LightCycler 480 software release 1.5.1.62 (Roche). Primers for amplification of the genes of interest were designed using the PrimerQuest software (Integrated DNA Technologies Inc., http://eu.idtdna.com/Primerquest/Home/Index; Table S5). Upon comparison with *PP2A*, *UBQ10* and *GTPb*, the expression levels of the actin gene (*Manihot esculenta actin‐7*, Manes.12G150600) resulted as the most stable in our experimental condition; therefore, actin was used as a reference gene.

### 2.3 Data analysis

Raw data from RT‐qPCR experiments were analysed by LightCycler® 480 software (v 1.5.1.62). Tukey test (*p *< 0.05 and <0.01, as reported in each figure) was used for multiple pairwise comparisons while pairwise comparison was made by Students' test (*p *< 0.05 and <0.01, as reported in each figure). The software IBM SPSS v19 was used for statistical analysis. Values of metabolites referring to 2 years were subjected to the analysis of variance (ANOVA) before statistical analysis. To analyse the relation between data from gene expression and metabolite content versus time in PFR/FR and PSR/SR, a regression analysis was carried out. The statistic *R* ^2^ (calculated by means of the IBM SPSS v19 software) was used to measure the goodness‐of‐fit for linear regression models, evaluating the strength of the relationship between our model (time) and the dependent variable (gene expression and metabolite content). Values >0.5 and <−0.5 indicate a positive and negative linear relation between independent (TPs) and dependent variables (gene expression level and metabolite content) at *p *< 0.01.

### 2.4 Transcriptomic data analysis

RNA samples (three biological replicates per tissue), depleted of ribosomal RNA (Ribo‐Zero rRNA Removal Kit Plant, Illumina), were sequenced with Illumina technology (Illumina NextSeq. 500) to obtain an average of 20 million paired‐end reads. We received sequencing raw files containing between 21 million and 60 million paired‐end reads. The raw reads were mapped with the STAR algorithm (Dobin et al., 2013) against the cassava reference genome (version 6.0). Mapping efficiency was between 67% and 87%. SAM files were converted to BAM files and sorted with the MergeSamFiles tool from the Picard package. Sorted BAM files were used by CuffLinks and CuffDiff algorithms (Trapnell et al., 2012) to calculate normalized fragments per kilobase per million reads (FPKM) using geometric means of fragment counts across all libraries (Anders & Huber, 2010) and the fold changes with corresponding *p *values. Two different data sets were produced: (i) root‐specific gene analysis; genes not expressed in leaves (FPKM ≤ 3 in all SIL and SOL samples) but in PFR/FR and/or in PSR/SR (FPKM ≥ 5 in at least one PFR/FR or PSR/SR sample) and differentially regulated between PFR/FR and PFR/SR with a foldchange of at least ±2, accepting a *p *≤ 0.15. (ii) Gene ontology (GO) analysis; genes differentially regulated between PFR/FR and PSR/SR (*p* ≤ 0.05) independent of the expression in the leaves.

### 2.5 GO‐based data analysis

The gene set of differentially expressed genes between PFR/FR and PSR/SR at each time point contained several hundreds to thousands of genes (PFR vs. PSR TP1: 558; PFR vs. PSR TP3: 2320; FR vs. SR TP7: 1553). Therefore, we used a GO strategy for data analysis to decipher the role of these genes in root development. In detail, the analysis was conducted through the following steps: (i) Each gene in the gene set was semantically compared with each gene in the same set using the concept developed in Tulipano et al. (2007) where the functional similarity was calculated as a distance to the closest common parent for each associated GO term. (ii) This distance allows clustering of all genes in gene sets and assignment of a descriptive GO term to each cluster: the head GO term. (iii) Each functional cluster contains a certain number of GO terms and is represented by a certain set of genes. We used this set of genes to calculate the level of over‐ or underrepresentation of the head GO term relative to its abundance within all GO annotations of all cassava genes (Tulipano et al., 2007). We did this calculation for all clusters at all three time points. (iv) Comparing the head GO terms among the different time points, we calculated the overrepresentation increase or decrease of each head GO term. (v) Each GO cluster term that increased or decreased its overrepresentation from one time point to another was ranked according to the level of overrepresentation of each GO annotation of each gene within the cluster. (vi) We calculated for each cluster a score as the sum of the scores of each term within the cluster, taking into account the probability of the term (see Tulipano et al., 2007) and the number of genes associated with this term; the larger the score the higher the overrepresentation of the functionality of the GO cluster. (vii) For each gene in each selected cluster, the expression and fold change data were extracted and visualized via a heatmap or a table. Custom codes were written in perl (v5.22.1). The GO database was locally installed as a mysql (Server version: 5.7.32‐0ubuntu0.18.04.1) dump (termdb) downloaded on 30 April 2020. The cassava GO annotations were extracted from the annotation file V6.1 (Mesculenta_305_v6.1.annotation_info.txt).

### 2.6 Metabolite analysis

Starch, sucrose, glucose and fructose were extracted from roots and determined using an enzymatic assay as described in Rosado‐Souza et al. (2019). Root samples were collected and analysed in three to four biological replicates over 2 consecutive years. Starch was purified using Percoll gradient and the size distribution of starch granules determined manually using ImageJ. For visualization, the starch granules were coated with platinum palladium and imaged with a Hitachi SU5000 SEM using a 5 kV electron beam.

### 2.7 X‐ray measurements

For the CT measurements, nine cassava plants were grown in pots with 200 mm diameter in sieved organic soil (Einheitserde Classic ED73; sieve grid 0.5 cm). One set (TMS‐IBA980581) of plants was transferred from tissue culture and the other set (TMEB419) of plants was grown from stakes. The plants were regularly scanned over a period of 4 months and grown under the conditions 50% humidity, with 28 and 25°C during the 12 h:12 h, light:dark, respectively.

The CT setup consists of a Comet 225 HP/11 X‐ray tube and a Fraunhofer XEye 2020 flat panel detector. The setup operates with a frame rate of 2.85 images per second in 16‐bits full frame mode and yields projection images of 2040 × 2048 pixels. For scanning, we used a tube voltage of 175 kV and a current of 4 mA with a 1 mm cupper pre‐filter. The illumination time of 350 ms was used for each of the 1600 projections during the 360° rotation resulting in a total scan time of 9 min for each plant. Thus, the effective dose to the plants during a single scan is around 200 mGy. The scans were conducted with an optical magnification of 1.14 resulting in a raw voxel size of 87.7 µm. The roots were segmented with an internal algorithm called RootForce developed by Fraunhofer IIS. With the segmented data, it is possible to calculate the root volume of the time‐resolved measurements.

## 3 RESULTS

### 3.1 SR development

**Figure 1:**
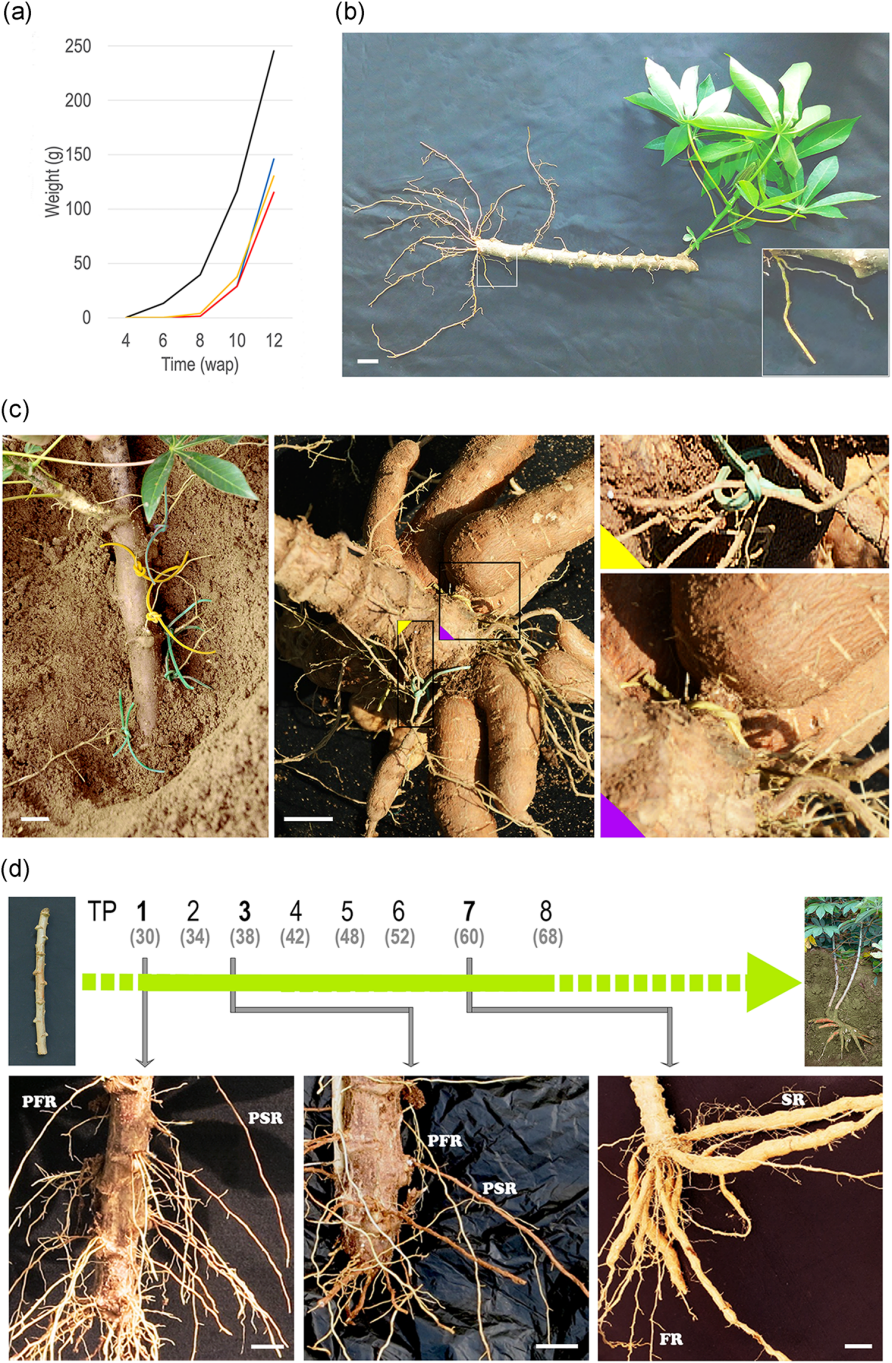
Storage roots development. (a) Fortnightly scoring of storage root weight per plant (four plants per time point). Four different genotypes with different yielding capacity were analysed: TMEB419 (black line); TMS‐IBA980581 (yellow line); TMEB693 (blue line); TMS‐IBA011412 (red line). (b) TMEB419 plant developing from a stem cutting. The inset shows magnified roots with distinct morphology. (c) Cassava roots labelled in yellow (PSR) and green (PFR) at four wap (first panel) and at harvest (second panel). Magnified sections of the image are shown in the insets. (d) Timeline of cassava plant development in the field from stake planting to harvest; monitoring time points (TP) one through eight are reported, with days after planting in brackets. Images of plants at TP1, TP3 and TP7 representing early, intermediated and evident storage roots development stages and showing exceptionally well‐distinguishable PFR and PSR are illustrated. Scale bars, 20 mm. PFR, potential fibrous roots; PSR, potential storage roots; wap, weeks after planting

Cassava roots, vegetatively propagated from stem cuttings (stakes) in field production systems, develop with a certain variability in time and consistency from stake to stake (Figure S1). Nevertheless, depending on the genotype and the environmental conditions, SR, arising from both nodes and the basal cut, become evident starting from 6 to 8 weeks after planting (wap, Figure 1a). At early developmental stages, the emerging and elongating roots appear morphologically similar. However, in some cases and upon careful observation, we noticed two different root morphologies never described before: (i) thinner, softer and lighter in colour and (ii) thicker, stiffer and darker (Figures 1b and S1). The morphological difference becomes more visible over time (between 4 and 8 wap, Figure 1d), albeit it is faintly discernible even shortly after root emergence from the stake (Figure S1). We named the first type ‘potential fibrous roots' (PFR) and the second type ‘potential storage roots' (PSR) and investigated whether the fate of PSR and PFR was indeed to develop into SR and FR, respectively. To this aim, we planted the earliest rooting genotype TMEB419 (Figure 1a), and at 4 wap we labelled putatively morphologically distinguishable PFR and PSR with rubber bands without uprooting the plants (Figure 1c, first panel). Four weeks later (8 wap), when SR bulking was clearly visible, we examined labelled root development (Figure 1c). The results obtained from two independent experiments including 47 roots (15 PFR and 32 PSR) and 70 roots (25 PFR and 45 PSR), respectively, revealed that none of the PFR‐labelled roots bulked, and 65% of the PSR‐labelled roots developed into SR. This indicates that not all PSR bulk: 35% remained thicker, darker and morphologically different from FR, but did not swell. To gain more insight into this physiological process, we performed a time‐course root monitoring experiment, sampling roots and leaves every 4 days, from stake planting until evidence of root bulking (Figure 1d). For a more in‐depth analysis, we selected three critical time points: TP1 (4 wap) when PFR and PSR are faintly discernible and their difference in size and morphology is not easily visible (Figure 1d, first panel and Figure S1), TP3 (6 wap) when PSR are thicker and darker but not yet swollen (Figure 1d, second panel), and TP7 (8 wap) when SR have bulked and are clearly distinguishable from FR (Figure 1d, third panel). Putatively morphologically different PSR and PFR (TP1 and TP3) and FR and SR (TP7) were collected, and the different tissue samples were used to investigate gene expression and metabolic changes occurring among them over time.

### 3.2 Functional gene expression analysis

To determine whether PFR and PSR are functionally different from one another and if they share similarities with their respective mature organs, we performed comparative gene expression profile analysis to investigate molecular differences over time. Paired‐end RNA sequencing (RNA‐Seq) was performed from samples of pooled SOL, SIL, PFR/FR, and PSR/SR, in biological triplicates across the three selected time points. RNA‐Seq‐derived transcript profiles were obtained, and the total abundance of each transcript was assessed after normalizing the different samples and adjusting the number of FPKM. In total, 29 463 transcripts (out of the 33 033 predicted genes) were detected in at least one tissue and one time point.

**Figure 2:**
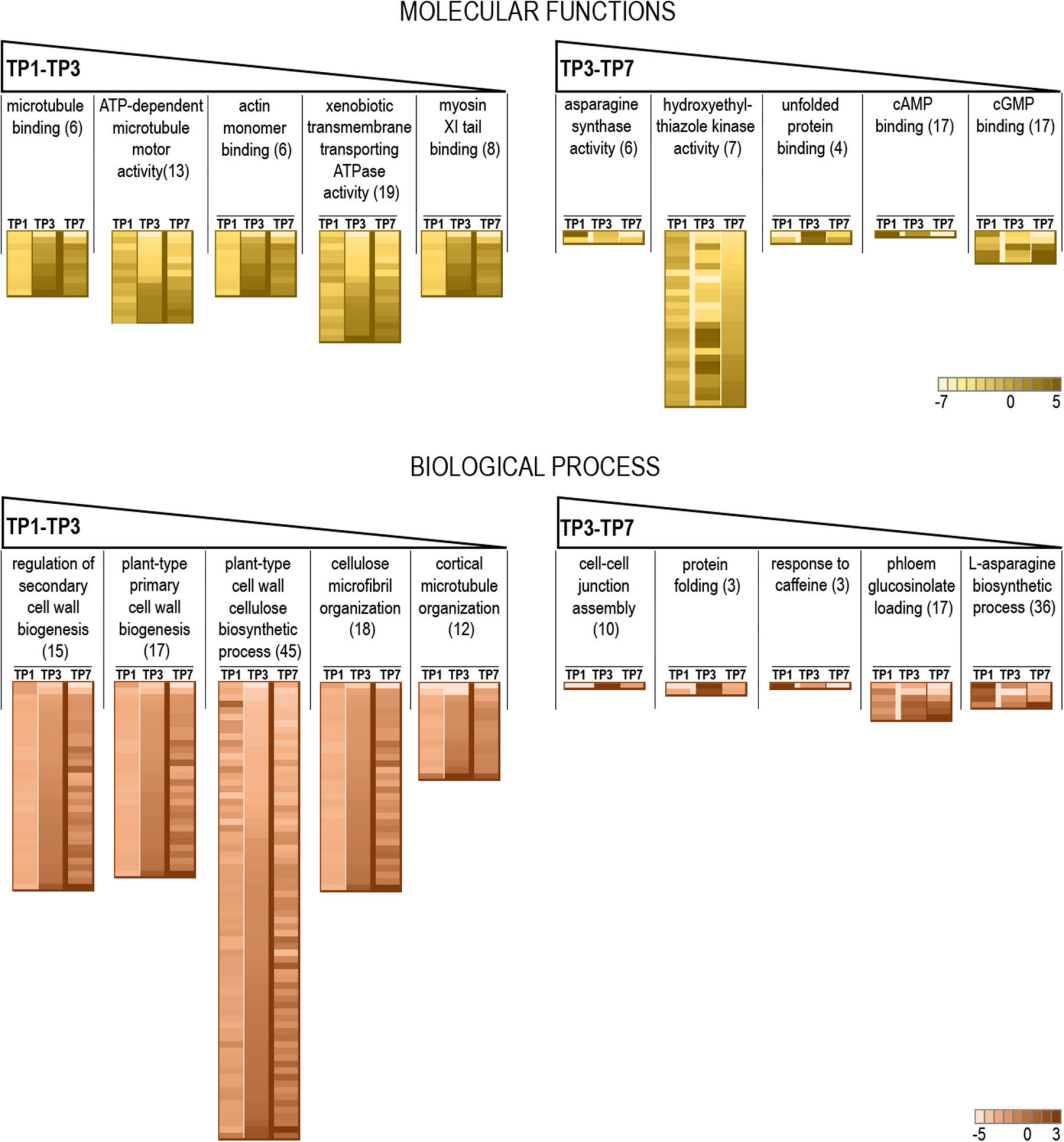
Functional gene expression analysis using GO. The five most significant clusters of over‐represented functionalities of the Molecular Function and the Biological Processes domains are given in order of decreasing importance (left to right) for the comparison TP1−TP3 and TP3−TP7. The heatmap below each cluster shows fold changes between FR and SR of the genes associated with the functional cluster differentially expressed at each TP. Variation in colour intensity corresponds to variations in fold change of expression values (the darker is the colour, the highest is the difference in gene expression in the two organs) as illustrated in the scales. The number of GO terms forming the specific cluster is reported in parenthesis. FR, fibrous roots; GO, gene ontology; SR, storage roots; TP, time point [Color figure can be viewed at wileyonlinelibrary.com]

To identify functions and processes specifically related to SR formation, we selected genes significantly (*p* < 0.05) differentially expressed between PFR/FR and PSR/SR for each of the three TP and used GO to cluster them (Tulipano et al., 2007). The obtained clusters contain genes with similar functionalities identified by similar GO terms and are ranked based on changes of GO representation between time points in each GO category (Figure 2 and Table S1). The expression fold change of the genes associated to each functional cluster was compared over the three TPs (Figure 2, heatmaps).

At early time points in the absence of formed SR (Figure S1), the GO analysis (TP3 vs. TP1 comparison) revealed the specific activation of molecular functions and biological processes related to cell growth and division in PSR relative to PFR. This included the remodelling of the cytoskeleton (indispensable for assembly of new cellular structures and organellar dynamics), cell wall biogenesis and remodelling, and membrane biogenesis (Figure 2 and Table S1). The expression of most of the genes relevant to the functions regulated in PSR between TP1 and TP3, remains largely unchanged until TP7, after root bulking (Figure 2, heatmaps).

On the other hand, the expression of another set of genes controlling functions and processes implicated in cell growth and differentiation (i.e., protein folding, cytoprotective unfolded protein response, amino acid synthesis and intercellular exchanges) and specifically regulated at TP3 in PSR becomes inversely regulated in SR at TP7, when root swell is appreciable (Figure 2 and Table S1).

Root thickening, mainly driven by increased production of vascular and parenchymatous tissues, relies on the activation of a number of specific genes. In particular, vascular formation is strongly regulated by the transcription factor gene family *VASCULAR‐RELATED NAC‐DOMAIN PROTEIN 1‐7 (VND1‐7)*. We observed that the *VND7* gene and the functionally related *XYLEM NAC DOMAIN 1* (*XND1*) gene, involved in transdifferentiation of various cells into metaxylem‐ and protoxylem‐like vessel elements (Kubo, 2005; Zhao et al., 2007), are both up‐regulated in cassava PSR/SR relative to PFR/FR (Table S2). On the other hand, genes regulating ectopic secondary cell wall formation in various tissues, such as *NAC SECONDARY WALL THICKENING PROMOTING FACTORs* (*NST1* and *NST2*) (Ko et al., 2007; Mitsuda et al., 2005; Zhong et al., 2006), *BEARSKIN2* (*BRN2*), and *MYB46* and *MYB83* (acting immediately downstream of *NSTs* and *VNDs*) (McCarthy et al., 2009; Zhong et al., 2007) are consistently down‐regulated in PSR/SR relative to PFR/FR (Table S2).

**Figure 3:**
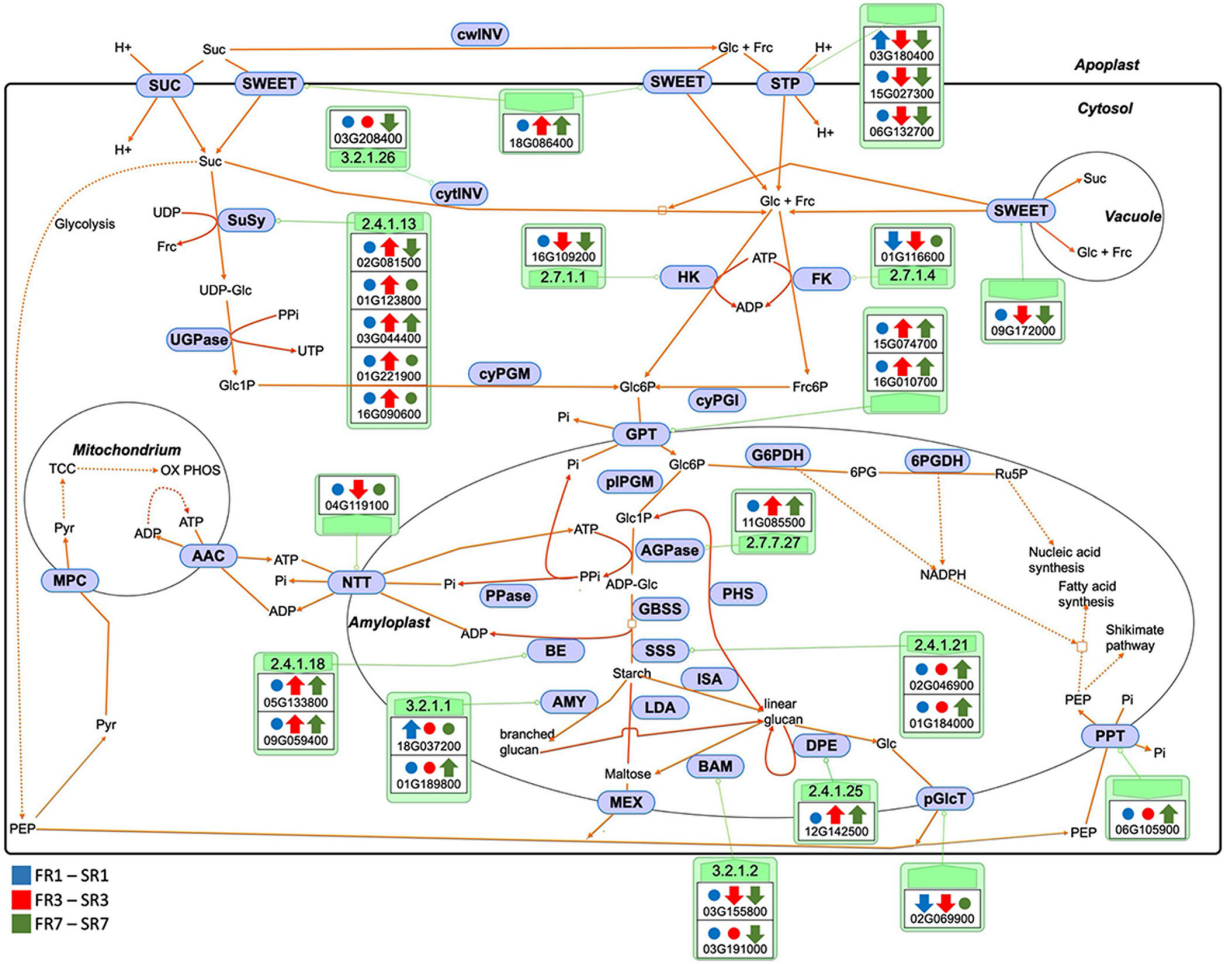
Sugar and starch metabolism. Major genes involved in sugar and starch metabolism regulated between PSR/SR and PFR/FR over the three TPs and located in different cellular compartments. The direction of the arrows represents increased (up) or decreased (down) level of expression of each gene in PSR/SR compared with PFR/FR at each TP (TP1, blue; TP3, red; TP7, green). Circles represent non‐regulated genes. FR, fibrous roots; PFR, potential fibrous roots; PSR, potential storage roots; SR, storage roots; TP, time point [Color figure can be viewed at wileyonlinelibrary.com]

As the main function of SR is starch accumulation, we monitored the regulation of the major genes supporting starch metabolism (Pfister & Zeeman, 2016) over the three time points and selected those differentially regulated between PSR/SR and PFR/FR at least in one TP (Figure 3).

The up‐regulation of a gene encoding SWEET transporter at the plasma membrane (Manes.18G086400, homolog to *AtSWET1*) and the down‐regulation of a SWEET transporter at the vacuolar membrane (Manes.09G172000, homolog to *AtSWET16*) at TP3 and TP7 suggest that imported sucrose and derived monosaccharides are probably not stored in the vacuole in SR, but rather directed towards starch biosynthesis or provide energy for root growth (Figure 3 and Table S3).

Downstream of SWEET, genes encoding sucrose synthases (*SuSy*, which catabolise imported sucrose), Glucose 6‐phosphate (Glc6P)/phosphate translocators (*GPT*s, which import glucose 6‐phosphate into amyloplasts), and ADP glucose pyrophosphorylase (*AGPase*, which synthesizes the substrate for starch synthesis) were up‐regulated in PSR compared with PFR at TP3 (and in most cases at TP7; Figure 3). We did not detect up‐regulation of cytosolic invertase (*cytINV*, which represents an alternative route for sucrose catabolism). Importantly, genes encoding Soluble Starch Synthase (*SSS*s) and Branching Enzymes (*BE*s)—both crucial for starch synthesis—were also up‐regulated in PSR/SR compared with PFR/FR at the later time points (Figure 3 and Table S3). Key genes involved in starch degradation such as β‐amylases (*BAM*s; which hydrolyse starch polymers to maltose) and *pGlcT* (which exports glucose derived from starch to the cytosol) (Lloyd & Kossmann, 2015) were down‐regulated in PSR/SR compared with PFR/FR. Interestingly, however, disproportionating enzyme (*DPE1*, which metabolises malto‐oligosaccharides) was up‐regulated. This enzyme is involved in starch degradation but may also play a role in starch biosynthesis since malto‐oligosaccharides are required to initiate starch polymer biosynthesis, and are also by‐products of the biosynthesis process (Ball & Morell, 2003; Colleoni et al., 1999; Tetlow & Emes, 2014).

This overtime gene expression comparison between PFR/FR and PSR/SR indicates that a clear activation of starch production in PSR starts at TP3 while genes involved in the differentiation of PSR/SR‐specific tissues are already active at TP1.

### 3.3 Root‐specific gene expression

To gain more insight into the transcriptomic signature of PSR/SR and PFR/FR and to identify potential root‐specific genes, we extracted, from the total gene expression data, genes with FPKM ≤ 3 in both leaf tissues (SIL and SOL) at all time points and considered them as genes not expressed in leaves. From this first selection, genes with an FPKM ≥ 5 in at least one root tissue (PFR/FR or PSR/SR) and in at least one time point (TP1, TP3, or TP7) were picked out as candidate root‐specific genes to be analysed for differential expression between PSR/SR and PFR/FR.

**Figure 4:**
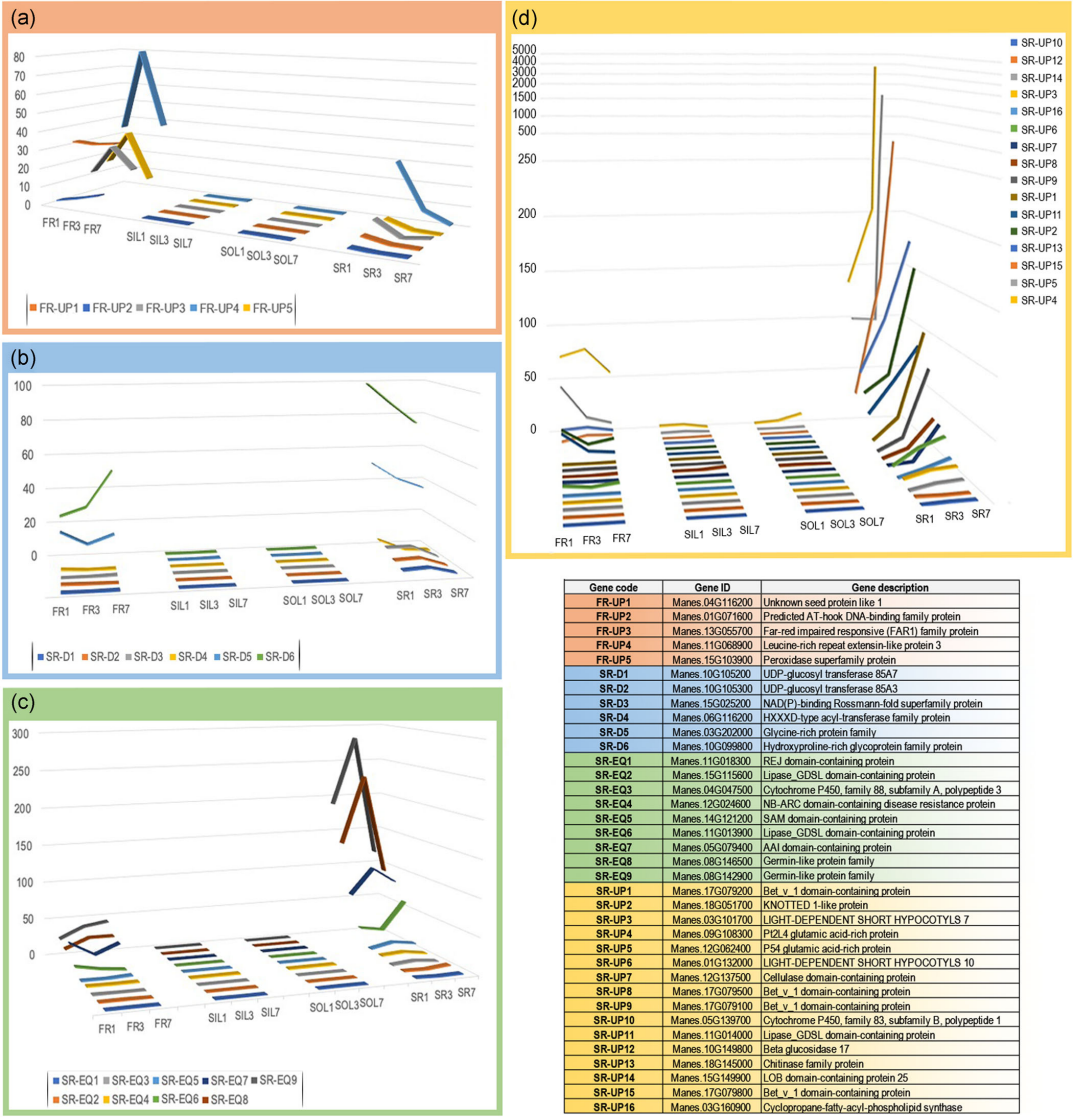
Root‐specific gene expression. Differentially expressed genes in four organs: PFR/FR; PSR/SR; SIL and SOL at three TPs. (a) Genes with increased expression in PFR/FR compared with PSR/SR; (b) genes with higher expression in PSR/SR compared with PFR/FR at TP1 and a decreasing trend of expression over time; (c) genes with a higher expression in PSR/SR compared with PFR/FR at TP1 and with undefined trend of expression over time; (d) genes with high expression in PSR/SR at TP1 and an increasing trend of expression over time. The *y*‐axis represents the relative gene expression calculated as fragments per kilobase of transcript per million mapped reads (FPKM). Corresponding Gene code, Gene ID and Gene description are listed in the enclosed table. FR, fibrous roots; PFR, potential fibrous roots; PSR, potential storage roots; SIL, sink leaves; SOL, source leaves; SR, storage roots; TP, time point [Color figure can be viewed at wileyonlinelibrary.com]

Based on these conditions, we identified five genes, all involved in cell growth and development, that were highly expressed in PFR/FR and lower in PSR/SR (Figure 4a and Table S4). The decreasing expression of such genes in PSR/SR over time suggests that they might be involved in the early development stages of PSR, but not during SR bulking (Figure 4a).

The comparatively larger number of genes higher expressed in PSR/SR compared with PFR/FR indicates the activation of a PSR/SR‐specific expression machinery for the development of this organ. Genes, active at TP1 in PSR and down‐regulated in the following TPs (SR‐D), are candidates for involvements in the initial steps of PSR/SR differentiation (Figure 4b and Table S4) and their different regulation in PFR/FR, particularly at TP7, indicates that developmental pathway might diverge very early between PSR/SR and PFR/FR. Among those genes whose expression in PSR/SR does not follow a well‐defined up‐ or down‐regulation trend over time (SR‐EQ), two encoding Lipase_GDSL domain‐containing protein known to be involved in the regulation of plant growth and development (Ling, 2008) show higher activity in developed SR (TP7) and down‐regulation over time in FR (Figure 4c and Table S4, SR‐EQ. 2 and SR‐EQ. 6). This group of genes also contained two encoding members of the germin‐like protein family, reported to participate, among other processes, to development, pollen formation, and response to abiotic and biotic stress (Dunwell et al., 2008), also highly expressed in PSR/SR, particularly at TP3 (Figure 4c, SR‐EQ. 8 and SR‐EQ. 9). A larger number of genes mainly involved in growth and development are specifically up‐regulated in PSR/SR over time (SR‐UP, Figure 4d and Table S4). Four genes in this group encode proteins of the Bet_v_1 domain‐containing superfamily of structurally related proteins comprising pathogenesis related and virus resistance proteins, and plant food allergens (SR‐UP1, SR‐UP8, SR‐UP9 and SR‐UP15) (Sinha et al., 2014; Song et al., 2020). Other genes in this group code for two cassava‐specific glutamic acid‐rich proteins involved in root development (de Souza & Carvalho, 2013; SR‐UP4 and SR‐UP5), a KNOTTED 1‐like protein favouring increase of cytokinin that promotes cell differentiation and SR morphogenesis (SR‐UP2; Liebsch et al., 2014; Meng et al., 2020; Utsumi et al., 2020), and two LIGHT‐DEPENDENT SHORT HYPOCOTYLS proteins (SR‐UP3 and SR‐UP6) up‐regulated in tobacco and Arabidopsis root growth and expansion stages (Bäumlein et al., 1991, Figure 4d).

### 3.4 FR and SR expression marker genes

**Figure 5:**
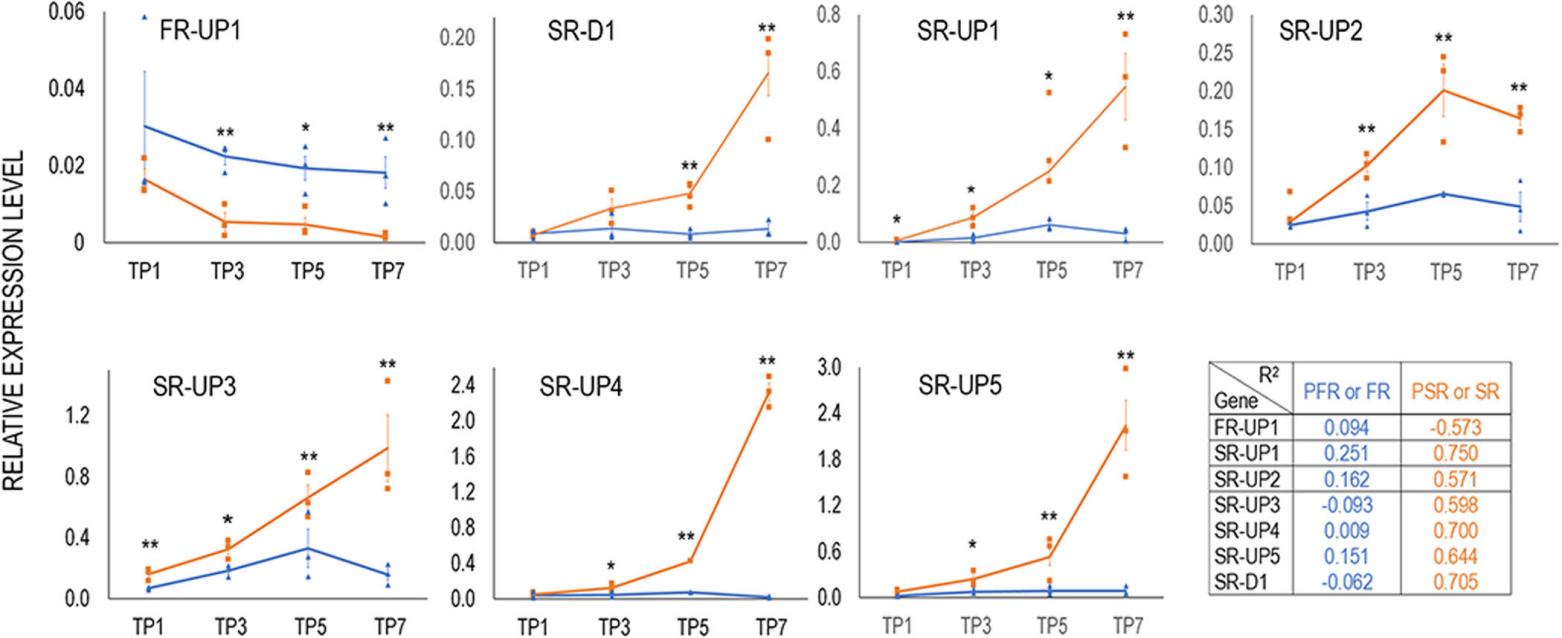
Gene expression in pools of PFR/FR and PSR/SR. Relative expression of seven selected genes differentially regulated between PSR/SR (orange line) and PFR/FR (blue line) measured at four different time points using RT‐qPCR. TP1, four wap; TP3, six wap; TP5, seven wap; TP7, eight wap. Data represent the average of three biological replicates±​​​​​​ ​SE, normalized by the actin gene as an internal control. Asterisks denote statistically significant difference according to two‐tailed Student's *t* test: *p < 0.05; **p < 0.01. In the panel, regression coefficient *R* ^2^ describes the trend of gene expression in PFR/FR and PSR/SR over time. FR‐UP1, unknown seed protein like 1; SR‐D1, UDP‐Glucosyl transferase; SR‐UP1, Bet_v_1 domain‐containing protein; SR‐UP2, knotted 1‐like protein; SR‐UP3, light‐dependent short hypocotyls; SR‐UP4, Pt2L4 glutamic acid‐rich protein; SR‐UP5, P54 glutamic acid‐rich protein. FR, fibrous roots; PFR, potential fibrous roots; PSR, potential storage roots; SR, storage roots; TP, time point [Color figure can be viewed at wileyonlinelibrary.com]

We sought to confirm the differential expression behaviour of the root‐specific genes identified and to investigate their potential use as expression markers specific for PFR/FR or PSR/SR in the initial stages of development. Therefore, we selected seven of the root‐specific genes (FR‐UP1, SR‐D1, SR‐UP1, SR‐UP2, SR‐UP3, SR‐UP4, SR‐UP5, Figure 4) and analysed their expression profiles in pools of PFR/FR or PSR/SR over four TPs (TP1, TP3, TP5, TP7, Figure 1d) using RT‐qPCR in an independent experiment. The expression profiles of the seven selected genes confirmed the results of the RNA‐seq analysis. FR‐UP1 is specifically up‐regulated in PFR/FR compared with PSR/SR where it constantly decreases over the TPs (negative *R* ^2^, Figure 5). Starting from TP3 the difference in expression between the root types becomes statistically significant and remains stable over time (*R* ^2^ = +0.094, Figure 5). SR‐D1 is up‐regulated in PSR/SR in the last two time points where the level of expression is significantly greater than in FR and increases during TPs (*R* ^2^ = +0.7). The expression of the SR‐UP genes increases over time in SR (*R* ^2^ > +0.5), faster for SR‐UP1, SR‐UP4 and SR‐UP5 then for SR‐UP2 and SR‐UP3 while the expression of the same genes in PFR/FR remains constantly low (*R* ^2^~0, Figure 5).

We tested whether these genes show similar expression behaviour also in other cassava genotypes, which is crucial to use them as molecular markers and potential biotechnological targets for crop improvement. The differential expression of FR‐UP1, SR‐UP3, SR‐UP4 and SR‐UP5 genes observed in TMEB419 could effectively discriminate between FR and SR of the genotypes TMS‐IBA090576, TMEB14 and KALESO at TP7, except for SR‐U4 in TMEB14 (Figure S2). These three genotypes feature high SR dry matter content (%dry weight/fresh weight) but contrasting performances in root size (RS), root number (RN) and root weight (RW) traits (Figure S2).

### 3.5 Metabolites analysis

**Figure 6:**
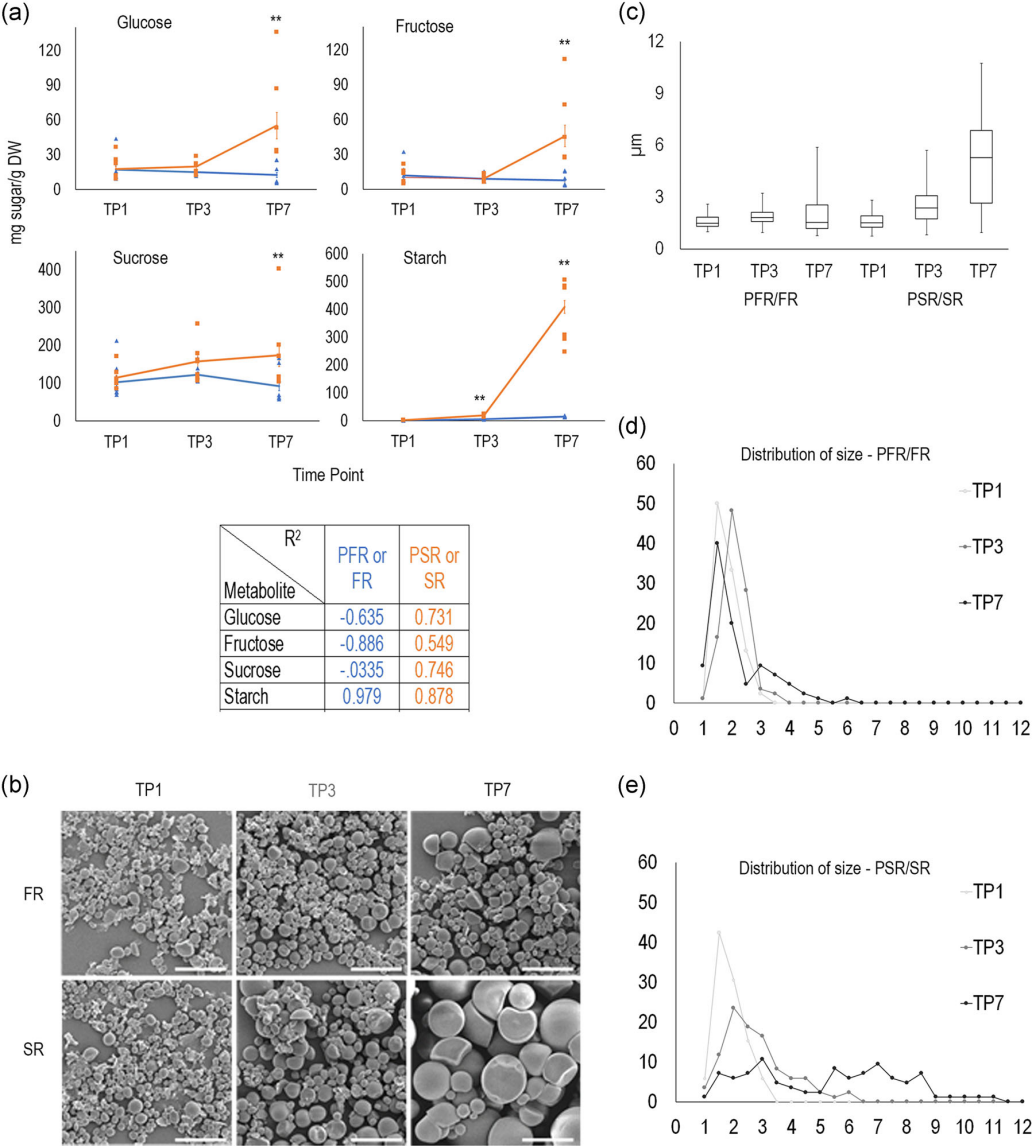
Physico‐chemical properties and quantification of root starch and soluble sugars. (a) Glucose, fructose, sucrose and starch content (mg g‐1 DW = dry weight) in pools of PFR/FR (blue line) and PSR/SR (orange line) at three time points (TP1, four wap, TP3, six wap, and TP7, eight wap). Data represent the average of seven biological replicates collected in 2 consecutive years. Asterisks indicate significant differences between the sugar content in PSR/SR and in PFR/FR at each time point according to two‐tailed Student's *t *test at *p* < 0.01. In the panel, regression coefficient *R* ^2^ describes the measured and compared relative amounts of soluble sugars and starch in PSR/SR and PFR/FR over time: +1 indicates a perfect positive linear relationship, −1 indicates a perfect negative linear relationship; values between 0 and 0.3 (0 and −0.3) indicate a weak positive (negative) linear relationship; the other values indicate a moderate positive (negative) relationship. (b) Scanning electron microscopy of the starch granules in PFR/FR and in PSR/SR at the three TPs. Scale bars, 10 µm. (c) Average of starch granules diameter in PFR/FR and in PSR/SR at the three TPs. (d) Distribution of starch granules size (diameter in µm) in (d) PFR/FR and in (e) PSR/SR at the three TPs. Counts, 84 granules per sample. FR, fibrous roots; PFR, potential fibrous roots; PSR, potential storage roots; SR, storage roots; TP, time point [Color figure can be viewed at wileyonlinelibrary.com]

Root growth is mainly dependent on carbohydrates transported via the phloem in the form of soluble sugars (principally sucrose) from source organs. Initially, planted cassava stakes will rely on the remobilization of stored sugars and starch until leaves emerge, mature into SOL and provide photoassimilates. Later, soluble sugars will also be used for starch accumulation in cassava SR. We measured and compared relative amounts of soluble sugars and starch in PFR and PSR at TP1, TP3, and FR and SR at TP7. Before SR bulking (TP1 and TP3), when both root types are rapidly developing, comparable amounts of soluble sugars were present in PFR and PSR (Figure 6a). At TP7, when SR are visibly swollen, and FR continue elongating, the soluble sugars content remained stable or slightly decreased in FR (negative *R* ^2^). In contrast, fructose, glucose and sucrose content increased in SR relative to TP1 and to FR (Figure 6a). This increase in soluble sugars presumably provides energy for continued PSR expansion and for starch biosynthesis in the bulking SR.

As expected, the magnitude of starch concentration correlates with SR swelling (Figure 6a). Starch is present in very low concentration in both PFR and PSR at TP1 and remained low in PFR/FR over time. In PSR/SR, starch content started to increase at TP3 and in bulked roots (TP7), starch had increased 40‐fold relative to FR (Figure 6a).

We used scanning electron microscopy (SEM) to visualise starch granule morphology in PFR/FR and PSR/SR over the different stages of root development. While granule shape appeared similar among all samples (Figure 6b), the granules size increased over time, with massive increases in PSR/SR (Figure 6c). In PFR/FR, most granules at all three TPs were small (~2 µm), but the frequency of bigger granules increased at the latter TPs (Figure 6d). In PSR/SR, the granules diameter significantly increases over time (Figure 6e). At TP1, all granules were small, as in PFR/FR. At TP3 a significant fraction of the granules had diameters >3 µm. Interestingly, at TP7, two populations of granules could be identified: smaller granules with diameters between 1 and 5 µm and granules with diameters between 5 and 10 µm (Figure 6e).

### 3.6 Gene expression and carbohydrate levels in single roots of single plants

Our results suggest that sugar levels do not change significantly between PFR and PSR before root bulking (TP7) whereas differential gene expression in the two organs occurs already at TP1, far earlier than the onset of morphological organ changes.

**Figure 7:**
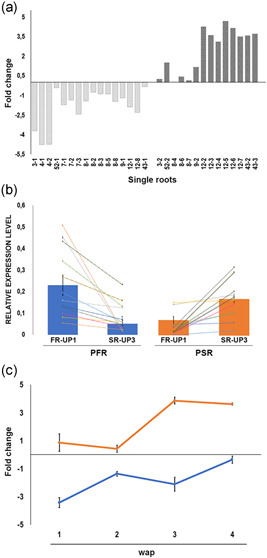
Single root analysis from one through four wap. 1‐wap‐old: plant 3, roots 1 and 2; plant 4, roots 1 and 2; plant 52, roots 1 and 2. 2‐wap‐old: plant 7, roots 1, 2 and 3; plant 8, roots 1 through 8; plant 9, roots 1 and 2. 3‐wap‐old: plant 12, roots 1 through 8. 4‐wap‐old: plant 43, roots 1, 2 and 3. (a) Fold change of expression of FR‐UP1 with respect to SR‐U3 calculated on expression values obtained by RT‐qPCR. Codes on the *x*‐axis represent specific plant−root combinations (plant number−root number). (b) Average ±SE expression values of FR‐UP1 and SR‐U3 measured in single roots by RT‐qPCR and normalized by the actin gene as an internal control. PFR and PSR represent all roots included in the negative and positive fold change groups identified in (a) respectively. Coloured lines connect the expression values of the two genes in each single root. (c) Average ±SE of fold change of expression of FR‐UP1 with respect to SR‐U3 in single roots of the same age. PFR, potential fibrous roots; PSR, potential storage roots; wap, week after planting [Color figure can be viewed at wileyonlinelibrary.com]

Seeking further confirmation that PSR and PFR differ early in their development, we selected two genes (FR‐UP1 and SR‐UP3) having contrasting levels of expression in PSR and PFR (Figure 5). We determined their expression levels in individual roots of single cassava plants, weekly from root emergence from the planting stem (1 wap) until 4 wap (TP1). We calculated the fold change values of FR‐UP1 with respect to SR‐U3 for each and all collected roots. According to the fold change, the young roots analysed split into two statistically significant distinct groups (two‐way ANOVA *F* = 52.294, *p* < 0.001) of opposite sign (Figure 7a). Consistently with the observations made in root pools at later time points (Figure 5), the expression values of FR‐UP1 and SR‐UP3 are inversely correlated in the two root groups thus supporting that these groups indeed represent PSR and PFR (Figure 7b). The fold change expression values plotted over the first weeks (1 through 4) after planting indicate that the trends of expression of the two genes in the two root types initiate at root emergence and continue over time until root bulking (Figures 5 and 7c). Regression statistics were calculated for both genes: SR‐U3, *R* ^2^ = 0.612 with *p *= 0.00095 (Fisher's test); FR‐UP1, *R* ^2^ = 0.422 with *p *= 0.011878 (Fisher's test). While the level of expression of SR‐U3 constantly increases in PSR over time, in PFR the level of FR‐U1 slightly decreases, and the combination of the two gene expressions is confirmed as a good marker for root type discrimination since root emergence (Figure 7c).

**Figure 8:**
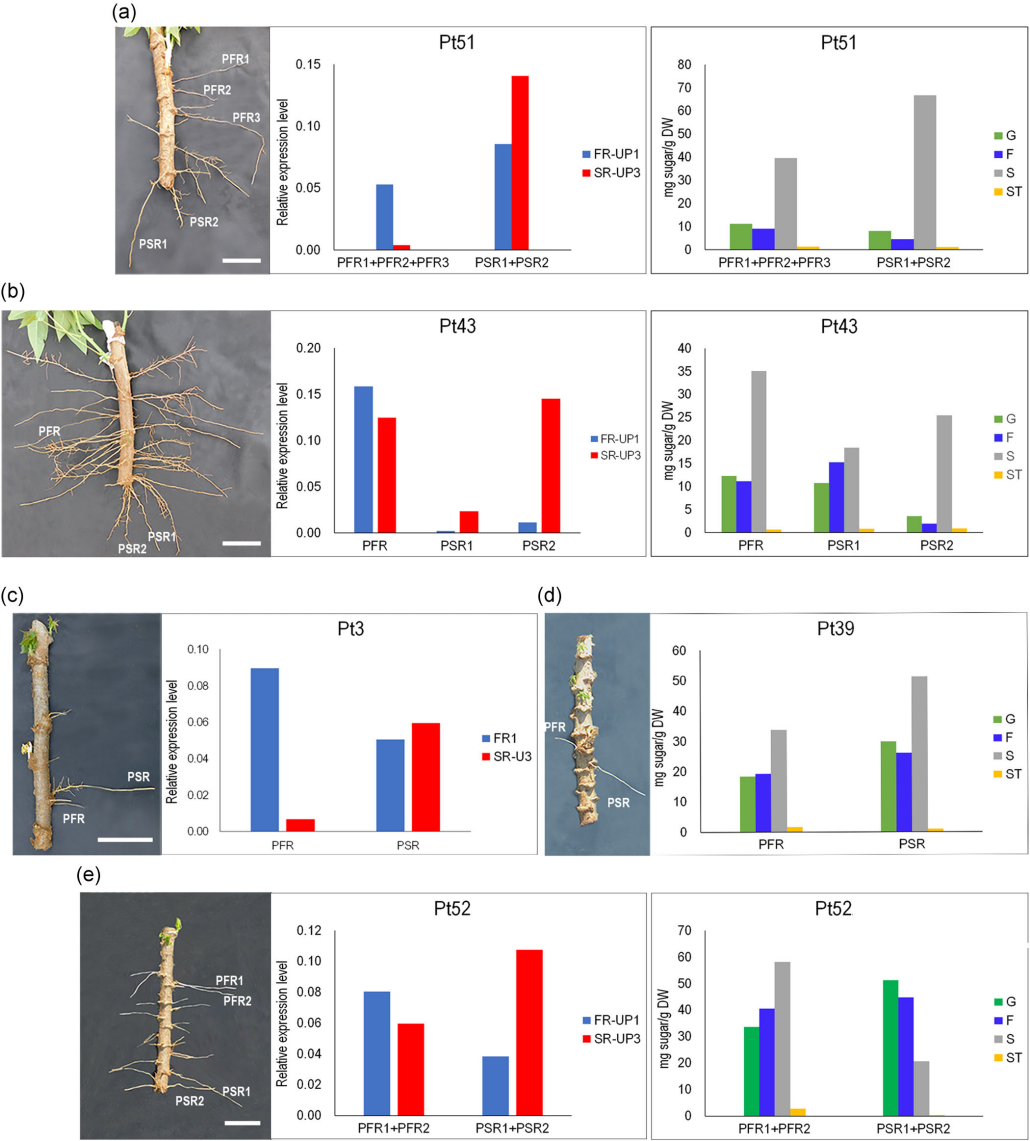
Gene expression and metabolites accumulation in single roots. Gene expression analysis of FR‐UP1 (unknown seed protein‐like 1) and SR‐UP3 (light‐dependent short hypocotyls) and metabolite (G: Glucose, F: Fructose, S: Sucrose, St: Starch) accumulation in roots of single plants (Pt). (a) Plant 51, root pools and (b) Plant 43, single roots. (c) Plant 3, single root; (d) Plant 39, single root; and (e) Plant 52, root pools. Plants in (a) and (b) were uprooted four wap and plants in (c), (d) and (e) were uprooted one wap. Corresponding pictures of each plant are shown. Normalization for RT‐qPCR was performed using the actin gene as an internal control. Scale bars, 50 mm [Color figure can be viewed at wileyonlinelibrary.com]

In roots showing an appreciable morphological difference between PFR and PSR, gene expression values, soluble sugars and starch content were measured and correlated in more detail. In plants uprooted 4 wap (equivalent to TP1, Figure 1d) in pools of two to three roots (Plant 51, Figure 8a) or as single roots (Plant 43, Figure 8b), relative up‐regulation of FR‐UP1 in PFR and of SR‐UP3 in PSR, as well as negligible starch accumulation and comparable soluble sugar contents, confirmed the results previously obtained from root pools (Figures 5, 6, and 8) and their correlation with the two subtly different PFR and PSR morphology types. Importantly, we observed this correlation also in roots just emerging from planting stakes (1 wap). The analysis of root samples collected from three distinct 1‐wap‐old plants confirmed a similar metabolite accumulation pattern in roots of Plant 39 and Plant 52 (Figure 8d, e), and the differential gene expression in roots of Plant 3 and Plant 52 (Figure 8c, d). Taken together, the observations made in single roots at early time points reveal that PSR and PFR are characterized by different gene expression profiles and morphological characteristics (increasingly visible overtime) since their emergence from the planting stake, but comparable soluble sugar and starch content at early developmental stages.

### 3.7 Non‐destructive analysis of cassava root apparatus development

**Figure 9:**
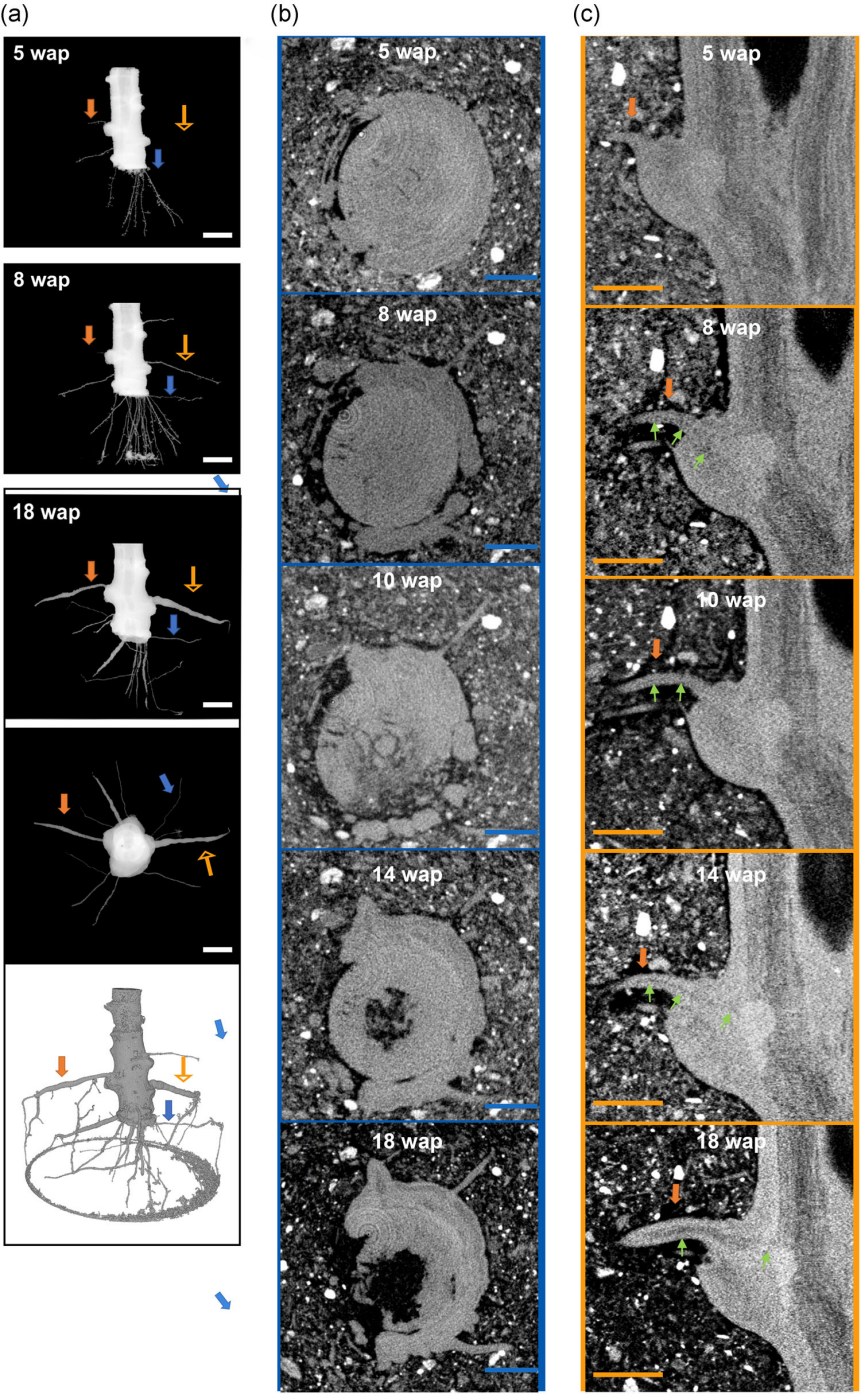
Non‐destructive underground cassava plant monitoring. Time course 3D segmented data of the underground growth of a cassava plant from stem planting to SR formation. (a) Longitudinal and transversal sections, and 3D reconstruction of a rooting stem cutting. Time resolution of two example roots: (b) PFR/FR, and (c) PSR/SR. Solid orange arrows indicate PSR/SR and blue arrows indicate PFR/FR illustrated in the time resolution. Hollow orange arrows indicate root illustrated in Figure S3. Green arrows point at the low‐density channel. In the images, light colour corresponds to high density. Scale bars, 10 mm. FR, fibrous roots; PFR, potential fibrous roots; PSR, potential storage roots; SR, storage roots [Color figure can be viewed at wileyonlinelibrary.com]

To gain more insight into the development process of the cassava root apparatus, non‐destructive X‐ray CT measurements were taken over a 13‐week time course in pot‐grown plants. Figure 9a depicts the 3D segmented data of a cassava plant showing underground growth from stake planting to SR formation.

Furthermore, time resolution of three example roots (one PFR/FR and two PSR/SR) is shown as virtual cross sections (Figures 9b, c and S3). Starting from 4 days after emergence (0.5 mm root diameter), we observed the presence of an inner channel‐like structure in some roots, illustrated by a drop in density (measured via the reconstructed attenuation coefficient and visible as dark regions) in the middle of the root, contiguous with the secondary vascular tissues of the stem (Figures 9c and S3). Shortly after root emergence, we detected the channel structure in 2−3 roots per plant while it was not visible in the remaining (3−5) roots of each analysed plant. None of the roots in which we could not see the darker channel structure turned into PSR/SR during the observation time (Figure S4), while in all swell SR the channel structure was detectable since emergence and enlarged continuously with root thickening (Figures 9 and S3). However, not all roots that displayed an inner channel swelled into an SR, which is consistent with our observation that not all roots visible scored as PSR swelled, despite being thicker and darker than roots scored as PFR. Interestingly, upon cutting of all formed SR from the planted stem, swelling reinitiated in the roots containing channel structure (PSR) at a belowground growth rate similar to that measured before SR cutting (Figure S5). When both SR and PSR (i.e., all channel‐containing roots) were physically cut off, we measured significantly longer time until plant roots started bulking again, and it occurred at an initial growth rate lower than before the cut (Figure S5). We never observed SR developing from PFR/FR left attached to the stem; therefore, bulking initiated from newly emerged PSR. These results indicate that PSR have the potential to start cell proliferation and starch accumulation so as to turn into SR, and in their absence PFR/FR (roots without channel structure) will not turn into SR.

## 4 DISCUSSION

Our results challenge the established hypothesis that cassava yield, and potentially that of other root crops, depends on a developmental change in the fate of a subset of adventitious roots into SR. This hypothesis was based on the fact that histological differences and starch accumulation become detectable only shortly before SR start to swell. Comparing cassava roots over time until bulking began, we provide strong evidence that PSR/SR belong to a functionally different root type from FR, and are already committed to their developmental fates at their appearance on the planted stake. We show that PSR and PFR are anatomically and morphologically different since their emergence, and while at this stage their respective physical characteristics are slightly and occasionally visible, X‐ray CT illustrates a different anatomical structure (the inner channel structure), to our best knowledge, never described before at SR emergence of any root crop. Besides, gene expression analysis shows that functions related to cell division and parenchymatous tissue proliferation, essential for root swelling, are up‐regulated in PSR, in contrast to PFR, before morphological differences become manifest. When soluble sugars increase and starch accumulation becomes measurable in PSR (6 wap), the gene expression patterns imply that intense preparatory histological and biochemical modification have already occurred, reaching a steady state or in some cases even starting to be down‐regulated again.

SR swelling is the consequence of intense starch synthesis in specialized parenchymatous root tissue, specific to a subset of emerging roots that follow a different rhizogenic process than FR. We show it is unlikely that there is a signal that triggers significant starch accumulation in FR after emergence and their differentiation into SR. However, it does seem that there is a mechanism—potentially involving a shoot‐derived signal—that determines whether a PSR initiates starch synthesis and bulking or not. Indeed, not all PSR accumulate starch, and non‐swollen PSR are always observed in cassava plants until harvest. Interestingly, Lowe and Wilson (1974) described three morphologically different root types in sweet potato: thin white FR (<5 mm), thick pigmented SR, and thick pigmented pencil roots (5−15 mm) that do not form SR and resemble the non‐swollen PSR here described. By continuous monitoring of the root development over time with CT, we provide evidence of predetermination of SR and FR by showing that roots missing the inner channel, presumed to be xylemic parenchyma cells deputed to starch accumulation in mature SR (Chaweewan & Taylor, 2015), cannot swell, while existing PSR (containing the channel) can immediately bulk and accumulate starch. Therefore, PSR retain their ability to accumulate starch, which can be triggered upon certain circumstances also at later plant developmental stages upon activation of xylemic parenchyma differentiation, resulting in enlargement of the channel. As confirmed by our root labelling results (35% PSR not bulking) while a number of PSR are formed in a cassava plant, only some of them undergo the process of starch accumulation probably triggered by a signal yet to be uncovered.

Our observation of contiguous connection of the root and stem secondary xylem, previously only reported in swollen SR (Chaweewan & Taylor, 2015), is consistent with a distinct rhizogenesis of PSR, possibly involving a direct extension of the secondary xylem tissue of the stem, which itself accumulates starch (up to 42% of dry mass, Wei et al., 2015). Therefore, the presence of stem‐derived secondary xylem in PSR and not in PFR could explain why the former root type is preset to immediately activate tissue differentiation and ultimately starch accumulation.

Our results also shed light on the debate over where on the planting stake SR origin. Our and previous observations (Lowe & Mahon, 1982) of SR arising from both nodes and (prevalently) the basal cut of stems planted either in the field or in large pots (≧30 cm diameter) definitely rebut the hypothesis that cassava SR only form at stem nodes (Chaweewan & Taylor, 2015). However, yield reduction observed upon increasing cassava planting density (Silva et al., 2013) indicates that space limitation hinders SR development, and suggests that the sideways growth of PSR emerging from nodes is favoured in small pots (12 cm) as compared with basal roots (Chaweewan & Taylor, 2015).

We illustrated that PSR of field cultivated plants can be occasionally discriminated from PFR by a darker and stiffer appearance becoming progressively visible over time, conferred by the accumulation of suberin, phenolic compounds and other metabolites when periderm replaces epidermis during root secondary growth (Campilho et al., 2020). This process must be activated earlier and more intensively in PSR compared with PFR. The down‐regulation of genes associated with lignification and up‐regulation of genes involved in differentiation of metaxylem‐like vessels, detected in young PSR, suggest that the thin channel structure measured by CT in PSR (and not in PFR) corresponds to the early establishment of xylem parenchyma cells, which only later become distinguishable as hallmarks of SR via staining and histological analysis (Chaweewan & Taylor, 2015).

An important new insight provided by our study is that differential expression of selected genes can effectively discriminate between PFR and PSR in several cassava genotypes already 1 wap. The availability of early selection markers will enable correct discrimination and comparison between the two root types and will be critical to shed additional light on the process of SR development and to accelerate breeding selection. While orthologues of these marker genes are generally involved in growth and development, except for *Pt2L4* (SR‐UP4), *P54* (SR‐UP5) and *Knotted 1‐like* (SR‐UP2), the role of the selected genes in cassava SR development and their potential application in biotechnological approaches to engineer high yielding cassava plants is yet to be elucidated. Identification of SR as a distinct root type from FR, with an inherent gene expression profile that determines its specific physiological properties, is a critical step towards understanding regulation of SR development. We trust that better understanding of cassava SR development can help to accelerate practical solutions to increase root crop yield and contribute to food stability for cassava farmers, most of whom are small‐holder farmers in low‐income countries ('Ending hunger: science must stop neglecting smallholder farmers', 2020).

## CONFLICTS OF INTEREST

The authors declare no conflicts of interest.

## Supporting information

Supporting information.

Supporting information.

Supporting information.

## Data Availability

High‐throughput sequencing data supporting the findings in this study have been deposited in the European Nucleotide Archive (ENA) at EMBL‐EBI under accession number PRJEB41121. Custom codes relative to the GO analysis are available upon request.
