# Supplementary material for: Set up from the beginning: The origin and early development of cassava storage roots
Source: Plant Cell Environ. 2022 Mar 30;45(6):1779–95. doi: 10.1111/pce.14300 (PMC9314696; doi:10.1111/pce.14300)
Supplement: Supplementary file 1 — Supporting information. [file PCE-45-1779-s003.docx]

## Set up from the beginning: the origin and early development of cassava storage roots (Carluccio AV. *et al.*)

## SUPPORTING MATERIAL


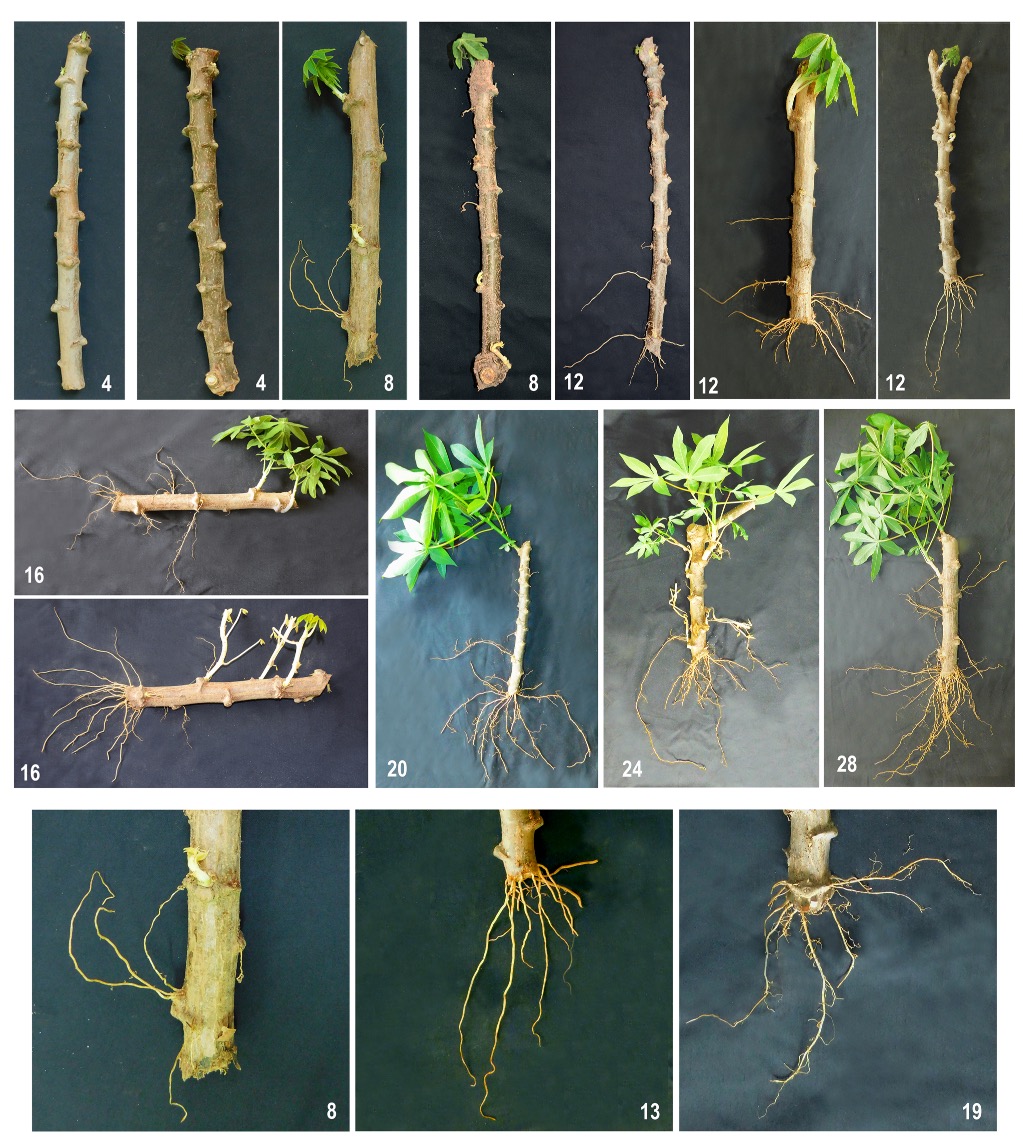


**Figure S1.** Stages of roots development in field-grown plants. Numbers refer to days after planting.

**
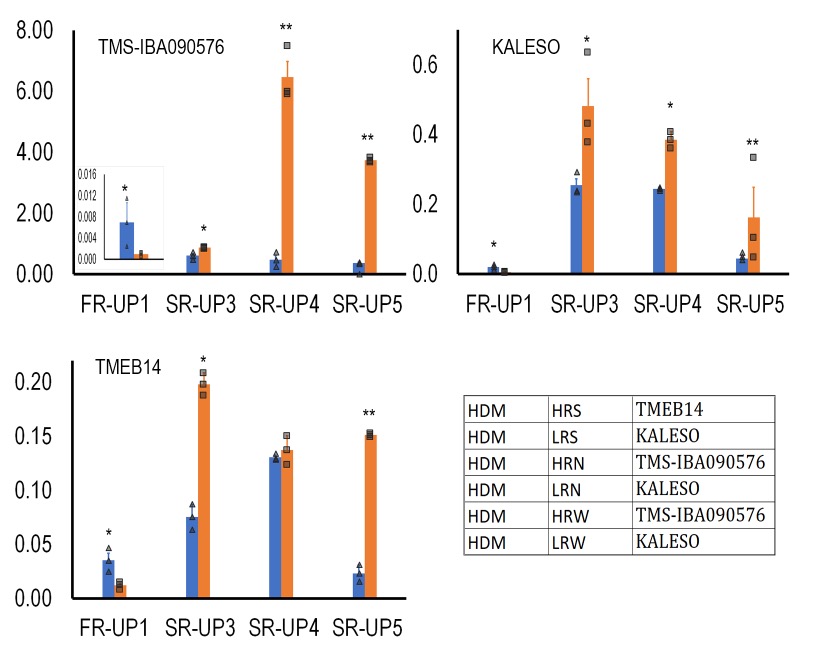
**

**Figure S2.** Relative gene expression of four genes differentially expressed between FR (blue plots) and SR (orange plots) in three different genotypes (TMS-IBA090576; Kaleso; TMEB14) at TP7, eight wap. Normalization was performed using the actin gene as an internal control. Each value is the average between three biological replicates + SE. Asterisks represent a statistically significant difference according to two-tailed Student’s *t* test; * and ** indicate P < 0.05 and P < 0.01, respectively. FR-UP1: Unknown seed protein like 1; SR-UP3: Light-dependent short hypocotyls 7; SR-UP4: Pt2L4 glutamic acid-rich protein; SR-UP5: P54 glutamic acid-rich protein. The three genotypes are scored for agronomic traits, HDM, High Dry Matter, H/LRS, High/Low Root Size, H/LRW, High/Low Root Weight, H/LRN, High/Low Root Number.


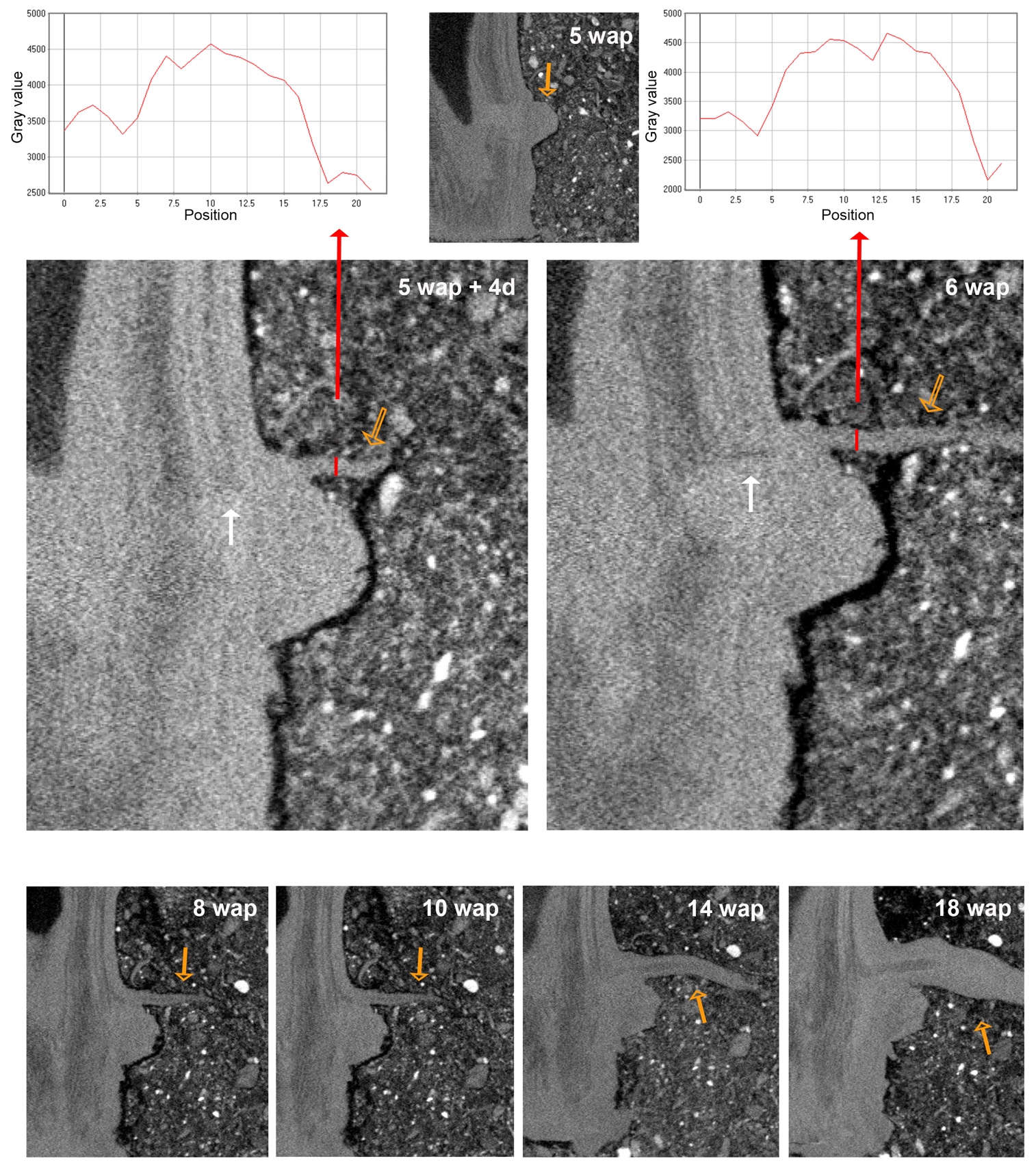


**Figure S3.** Time course development of a PSR indicated by an orange hollow arrow. At five wap, the root has not emerged yet. Within the following four days (d) the new root emerges from the stem; the extension of the stem secondary xylem towards the roots is shown by a white arrow. A small channel structure is depicted by the drop of density in the middle of the root illustrated in the diagram above both pictures (5 wap+4d: position ~8; 6wap: position ~12). At six wap, the density drop further increases and the connection with the stem is more visible (white arrow). From eight wap, the channel structure is clearly visible and continues enlarging overtime, linearly with the root diameter increase. Orange hollow arrows in Fig. 8a, indicate the position of this root on the stem and development of the same root overtime. In the images light colour corresponds to high density.

**
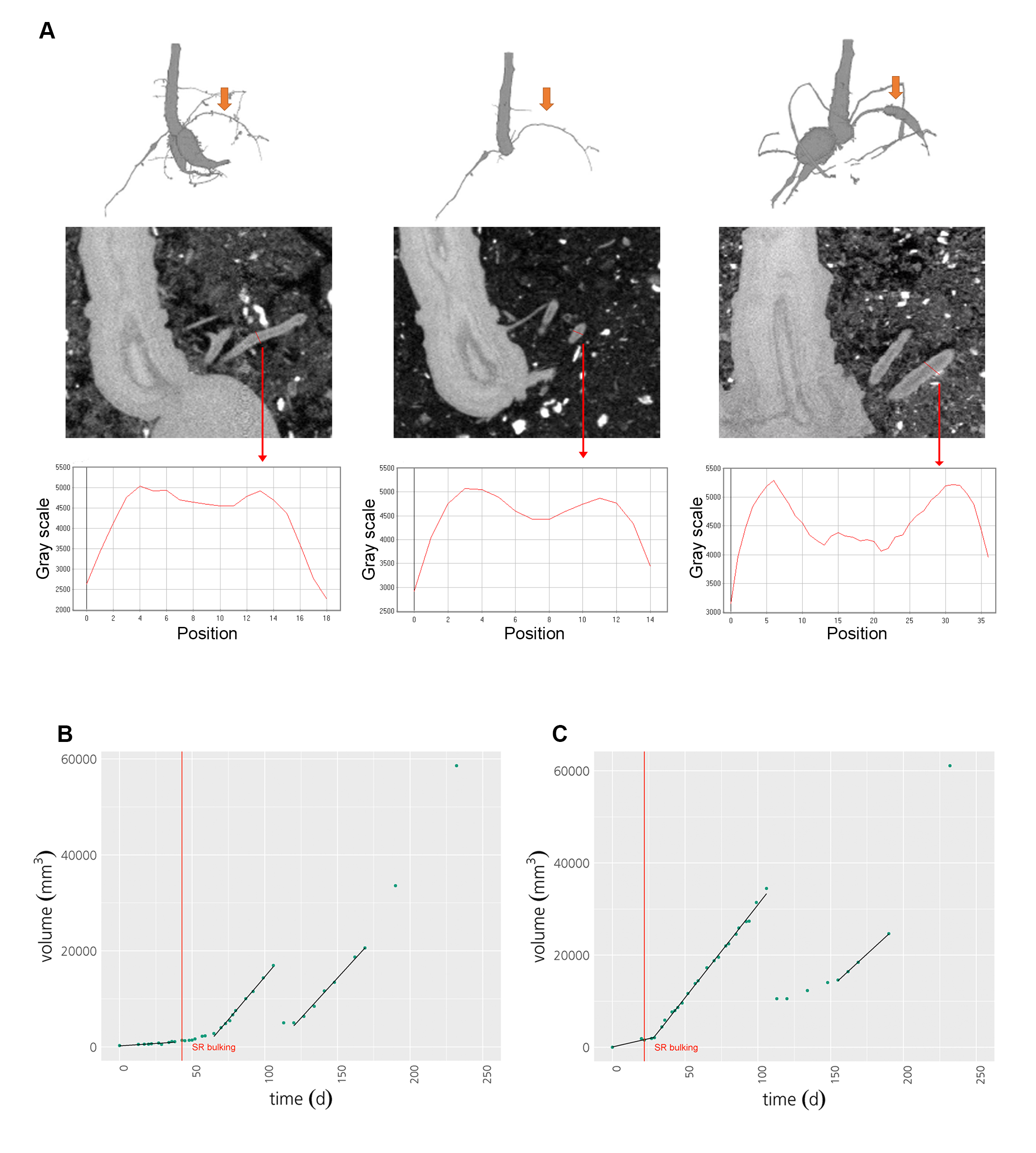
**

**Figure S4** Development of new SR after cut of existing ones. (a) Longitudinal sections of a cassava rooting stem (TMS-IBA980581) before (left panels) immediately after (middle panels) and five months after cutting (right panels) existing storage roots, as shown in the thumbnails above the pictures. Diagrams illustrate a drop in density (darker area) in the middle of the PSR/SR root indicated by an orange arrow in the picture. Monitoring of volume increase of the root apparatus before and after the cut of (b) all swelling SR, and (c) all roots with a channel structure contiguous with the stem secondary vasculature (PSR + SR).


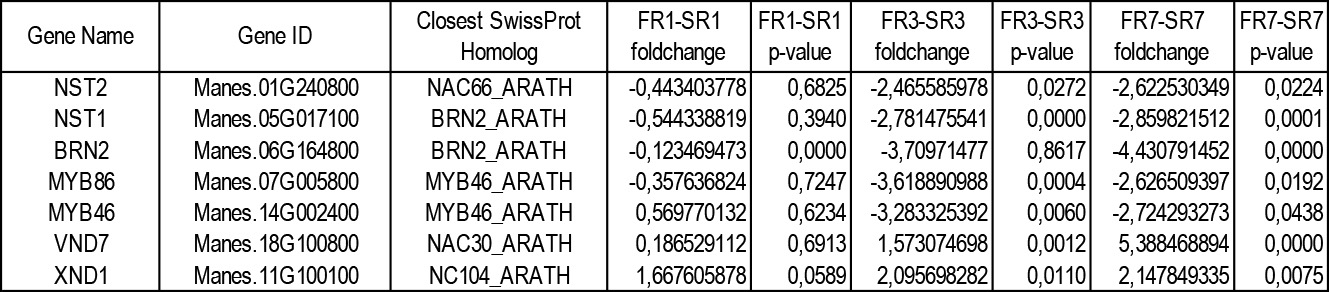
**Table S2.** Foldchange (FR vs. SR) and p-value of genes involved in root thickening.


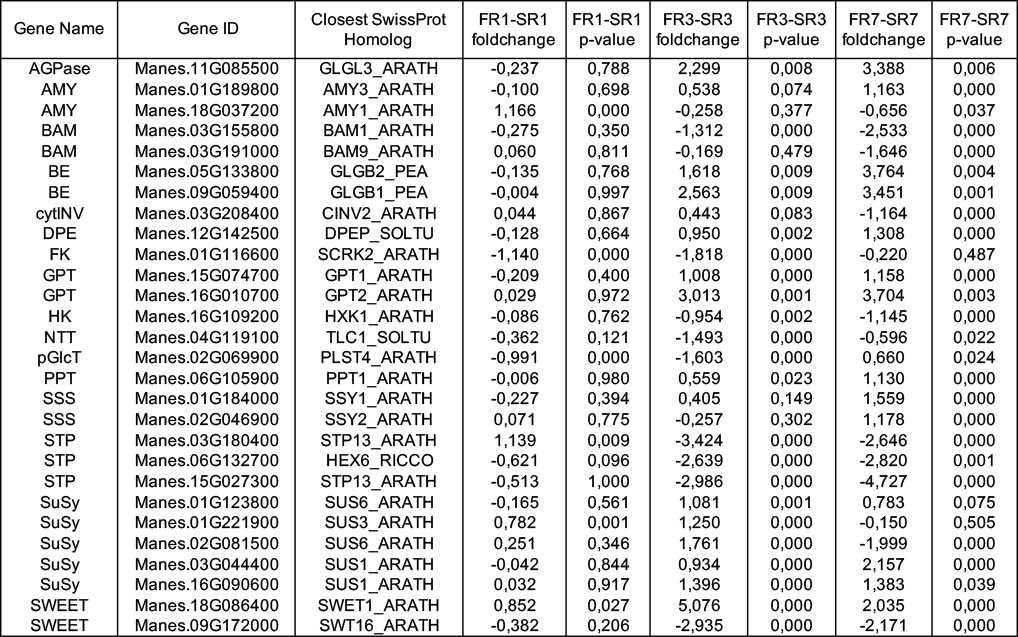
**Table S3.** Foldchange and p-values data (FR vs. SR) of genes involved in starch metabolism illustrated in Fig. 3.


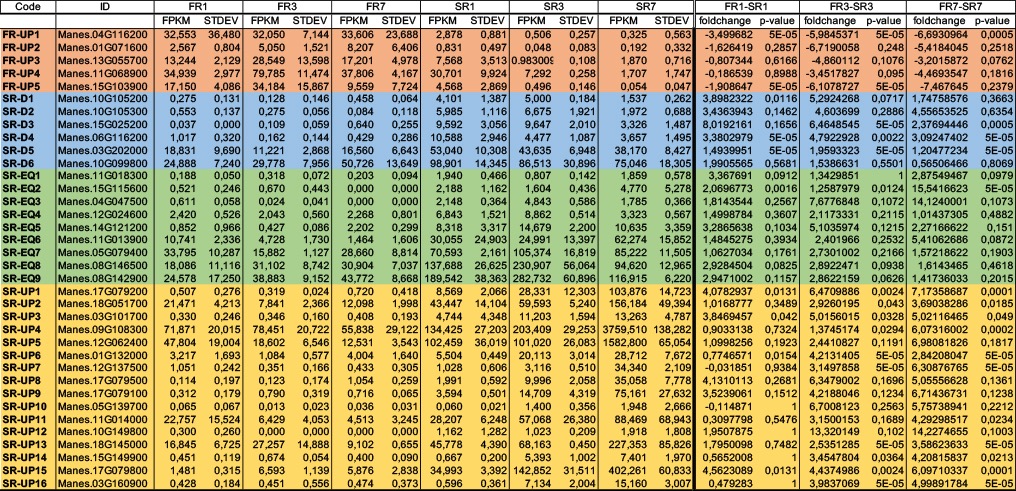


**Table S4.** Expression values (with standard deviation of the biological replicates) of potential storage root-specific genes and foldchange (FR vs SR) with relative p-values.

| **Gene Name** | **Manes** | **Primers** | **Amplicon size (bp)** |
| --- | --- | --- | --- |
| SR-UP1 | Manes.17G079200 | F: CGTTGGACCTTAGAATATGAGAAGA | 110 |
|  |  | R: CATTTCACGAGATGAGCAGAAAC |  |
| SR-UP2 | Manes.18G051700 | F: CCACACAAAGCAACGATCTTC | 114 |
|  |  | R: GTAAGCTCTTCGCGGTACTT |  |
| SR-UP3 | Manes.03G101700 | F: GCTCCGAGCTGCTTATGAA | 106 |
|  |  | R: CTTAGCTTGACAGTCCCTCAC |  |
| SR-UP4 | Manes.09G108300 | F: CAGCAGAGACCGTTGTTGA | 108 |
|  |  | R: CTGGAGTTTCCGATGCTGAT |  |
| SR-UP5 | Manes.12G062400 | F: CTGGTTCCTCCTGAGATGTTT | 106 |
|  |  | R: GAGGTGGTAGAGGTTGAAGAAG |  |
| FR-UP1 | Manes.04G116200 | F: TGCAGACTTTGTGTTCGACTA | 114 |
|  |  | R: GTAGAAGGATCCTGAATGGGAAA |  |
| SRD1 | Manes.10G105200 | F: TGTGCCATGTATCCTCATTTAT | 78 |
|  |  | R: TTCTTTCCATAGACTTGAACCC |  |
| Actin | Manes.12G150600 | F: TCCGTGACATGAAGGAGAAG | 113 |
|  |  | R: CTGACCATCAGGAAGCTCATA |  |
| PP2A | Manes.09G039900 | F: CCTGATGTTACCCGTAGAACACC | 109 |
|  |  | R: CCTCAACCACCTGAGCGTAAA |  |
| UBQ10 | Manes.07G019300 | F: TTGTGAAGACCTTGACTGGGA | 111 |
|  |  | R: GCTGATCCGGTGGAATTCCTTC |  |
| GTPb | Manes.09G086600 | F: CCGTGGAGCTATGGGTATTTT | 111 |
|  |  | R: TTCACATTGTCAGAAGCATGTTG |  |

**Table S5**. RT-qPCR primer sequences.
