# Supplementary material for: Set up from the beginning: The origin and early development of cassava storage roots
Source: Plant Cell Environ. 2022 Mar 30;45(6):1779–95. doi: 10.1111/pce.14300 (PMC9314696; doi:10.1111/pce.14300)
Supplement: Supplementary file 3 — Supporting information. [file PCE-45-1779-s002.docx]

## Set up from the beginning: the origin and early development of cassava storage roots

## (Carluccio AV. *et al.*)

**
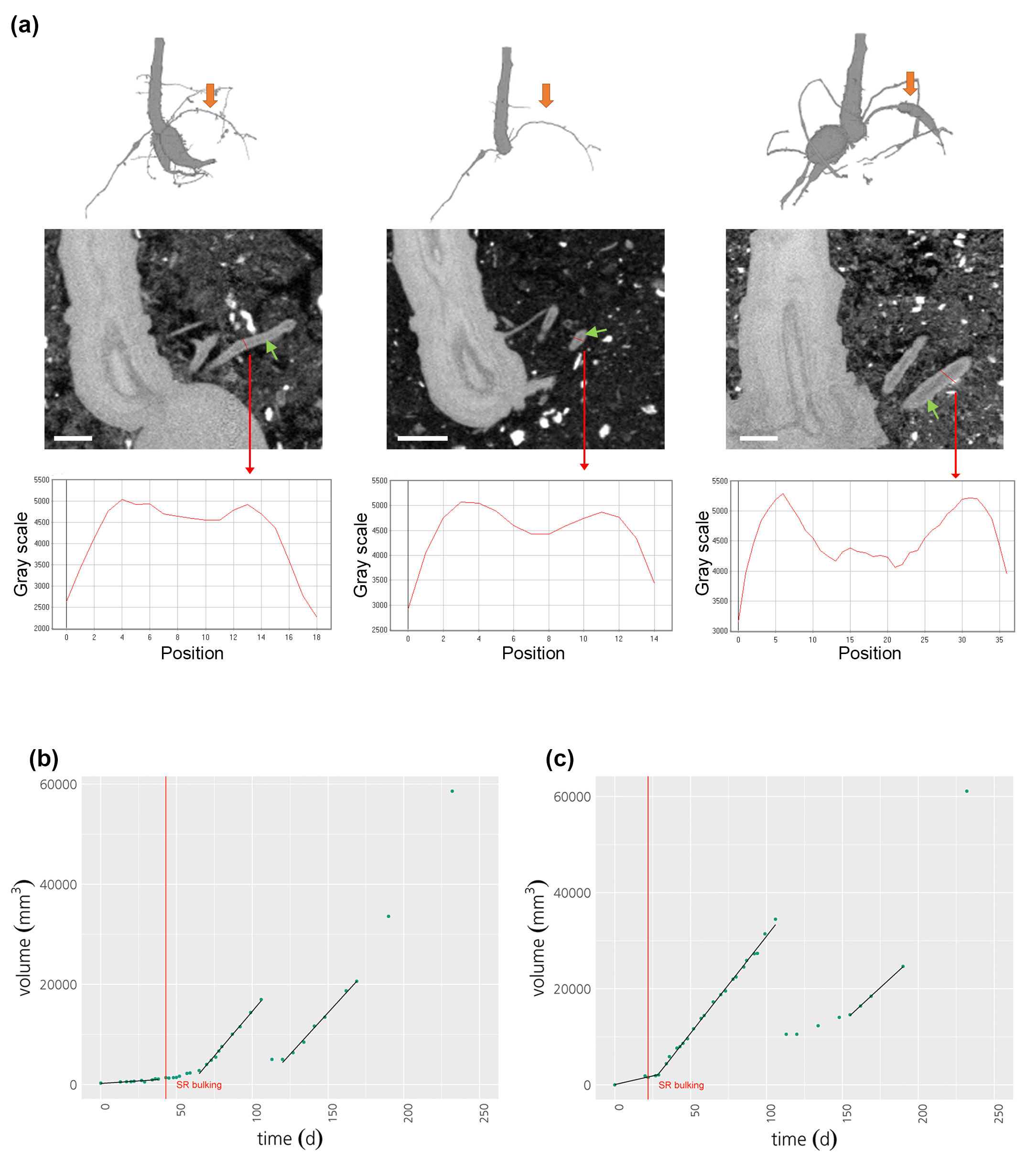
**

**Figure S5.** Development of new SR after cut of existing ones. (a) Longitudinal sections of a cassava rooting stem (TMS-IBA980581) before (left panels) immediately after (middle panels) and five months after cutting (right panels) existing storage roots, as shown in the thumbnails above the pictures. Diagrams illustrate a drop in density (darker area, pointed by a green arrow) in the middle of the PSR/SR root indicated by an orange arrow in the picture. Monitoring of volume increase of the root apparatus before and after the cut of (b) all swelling SR, and (c) all roots with a channel structure contiguous with the stem secondary vasculature (PSR + SR). Scale bars, 10mm
